# A young male with chronic nonproductive cough diagnosed with blastomycosis in China: a case report

**DOI:** 10.1186/s12890-020-01225-4

**Published:** 2020-07-11

**Authors:** Na Wang, Zhibing Luo, Shuangshuang Deng, Qiang Li

**Affiliations:** 1 Department of Respiratory, Shanghai East Hospital, Affiliated to Tongji University, No.150 Jimo Road, Pudong District, Shanghai, 200020 People’s Republic of China; 2 Department of Pathology, Shanghai East Hospital, Affiliated to Tongji University, No.150 Jimo Road, Pudong District, Shanghai, 200020 People’s Republic of China

**Keywords:** Blastomycosis, Next generation sequencing, Endobronchial ultrasound-guide sheath-transbronchial lung biopsy

## Abstract

**Background:**

Blastomycosis is a fungal infectious disease prevalent in North America and rarely reported in Asia. Misdiagnosis of malignancy and other infectious diseases were reported.

**Case presentation:**

A 24-years-old male patient presented with chronic non-productive cough of 4 months duration. He had been diagnosed with *Mycobacterium tuberculosis* infection and lung malignancy elsewhere and presented to us as the symptoms persisted. We offered him the biopsy under endobronchial ultrasound-guide sheath-transbronchial lung biopsy and sample specimen were sent for next generation sequencing analysis, returned as *Blastomyces Dermatitidis* infection. The patient was treated by itraconazole for 6 months, his symptoms decreased significantly and the CT scan showed resolution of the lesion.

**Conclusion:**

We shared a case of blastomycosis with delayed and difficult diagnosis and reviewed the knowledge regarding differential diagnosis and next generation sequencing technologies.

## Background

Blastomycosis is a systemic fungal infectious disease caused by the inhalation of the conidia of *Blastomyces dermatitidis*. It is endemic in North America along the Mississippi and Ohio River valleys, the Great Lakes, and the Saint Lawrence River. The disease, however, is rarely reported in Asia, with less than 20 cases reported in China, based on a research of Chinese literature databases [1]. Manifestations vary from asymptomatic or limited pulmonary involvement, to disseminated systemic infection in immunocompromised patients. Atypical or mild blastomycosis can be limited within the lung, and symptoms may mimic other infections such as *Mycobacterium tuberculosis* infection. Misdiagnosis of the lung malignancy from blastomycosis is, therefore, commonly reported. Herein, we described a case of atypical presentation of fungal pneumonia. Blastomycosis was diagnosed after we biopsied the lesion under endobronchial ultrasound-guide sheath-transbronchial lung biopsy (EBUS-GS-TBLB) and obtained next generation sequencing (NGS) analysis.

## Case presentation

**Fig. 1:**
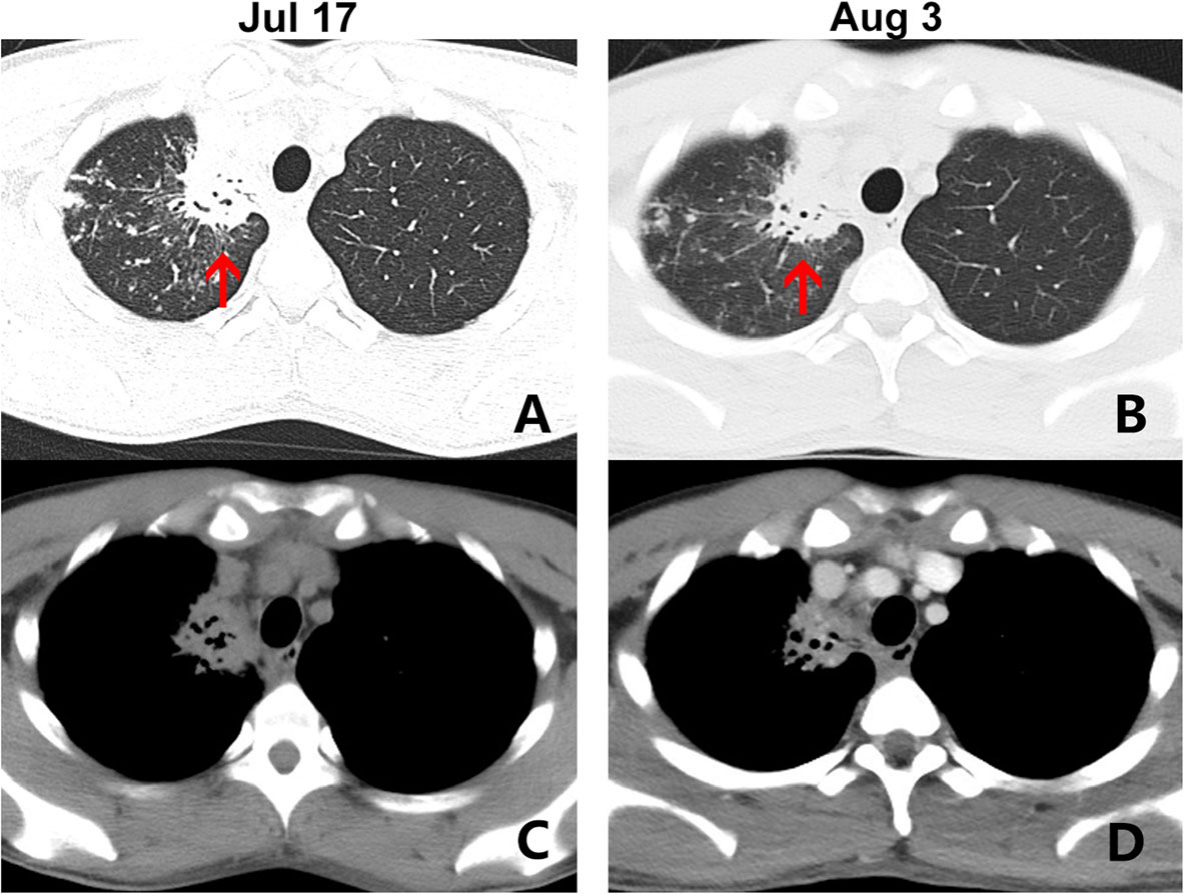
Chest CT scan of the lungs before admission. **a** A 30 × 37 mm-sized mass-like inflammatory lesion (red arrow) with air space located at the right upper lobe on lung window on July 17. **c** The same section on diaphragm window. **b** The same mass-like lesion (red arrow) with 32 × 28 cm size, irregular boarder and marginal long spiculation located at the right upper lobe on lung window on August 3. **d** Same section on diaphragm window

A 24-year-old previously healthy male was admitted to our hospital with a complaint of recurrent non-productive cough, which started 4 months earlier after a brief cold in April 2018. He recalled no accompanying symptoms (such as fever, sneezing, wheezing, sore throat, chest pain, shortness of breath, headache, and dizziness) and did not seek medical advice until early July 2018, when the cough had become persistent and white sputum production was noticed. Hence, he returned to China during the July summer holidays and had chest X-ray on July 13, 2018, which showed lung infection at the right upper lobe; thus, pulmonary tuberculosis was suspected. However, he had T-SPOT tests on the same day and was found negative. Chest Computed tomography (CT) scan was obtained on July 17, 2018, and reported as a mass-like inflammatory lesion, measuring 30 × 37 mm, with air space on the right upper lobe (Fig. 1). At the same time, his completed blood cells (CBC) showed normal white blood cells (WBC) count (6.3 × 10^9^/L), with normal differential leucocytes count including neutrophils (3.1 × 10^9^/L), lymphocytes (2.5 × 10^9^/L), monocytes (0.5 × 10^9^/L), eosinophils (0.1 × 10^9^/L), and basophils (0.00 × 10^9^/L), red blood cells (RBC) (4.81 × 10^12^/L) and platelet (280 × 10^9^/L) levels. Bronchoscopy was performed on July 20, 2018, in order to ensure precise diagnosis; collected broncho-alveolar lavage fluids (BALF) were sent for detection of Mycobacterium infection, and the results showed negative acid-fast stain and GeneXpert *Mycobacterium tuberculosis* DNA. Therefore, *Mycobacterium tuberculosis* infection was ruled out, and no anti-tubercular treatment was initiated. He repeated the chest CT scan with contrast on August 3, 2018 in another hospital due to persistence of symptoms. The second CT scan reported a mass measuring 32 × 28 mm on the right upper lobe, with an irregular spiculated border and moderate enhancement. Lung cancer with obstructive inflammation was, therefore, reported (Fig. 1). For precise diagnosis and management, the patient and his family approached us, seeking medical assistance. There was no change in the patient’s weight, appetite, sleep habits, and bowel movement. He is currently a graduate student at Illinois, USA. However, his travel history, as well as pet, home, and occupational exposures, were non-contributory.

Upon admission on August 7, 2018, he was afebrile and his blood pressure was 120/69 mmHg. Both lungs were clear on auscultation without rales or wheezing. No palpable lymph nodes, rash, or any skin lesion was present.

**Fig. 2:**
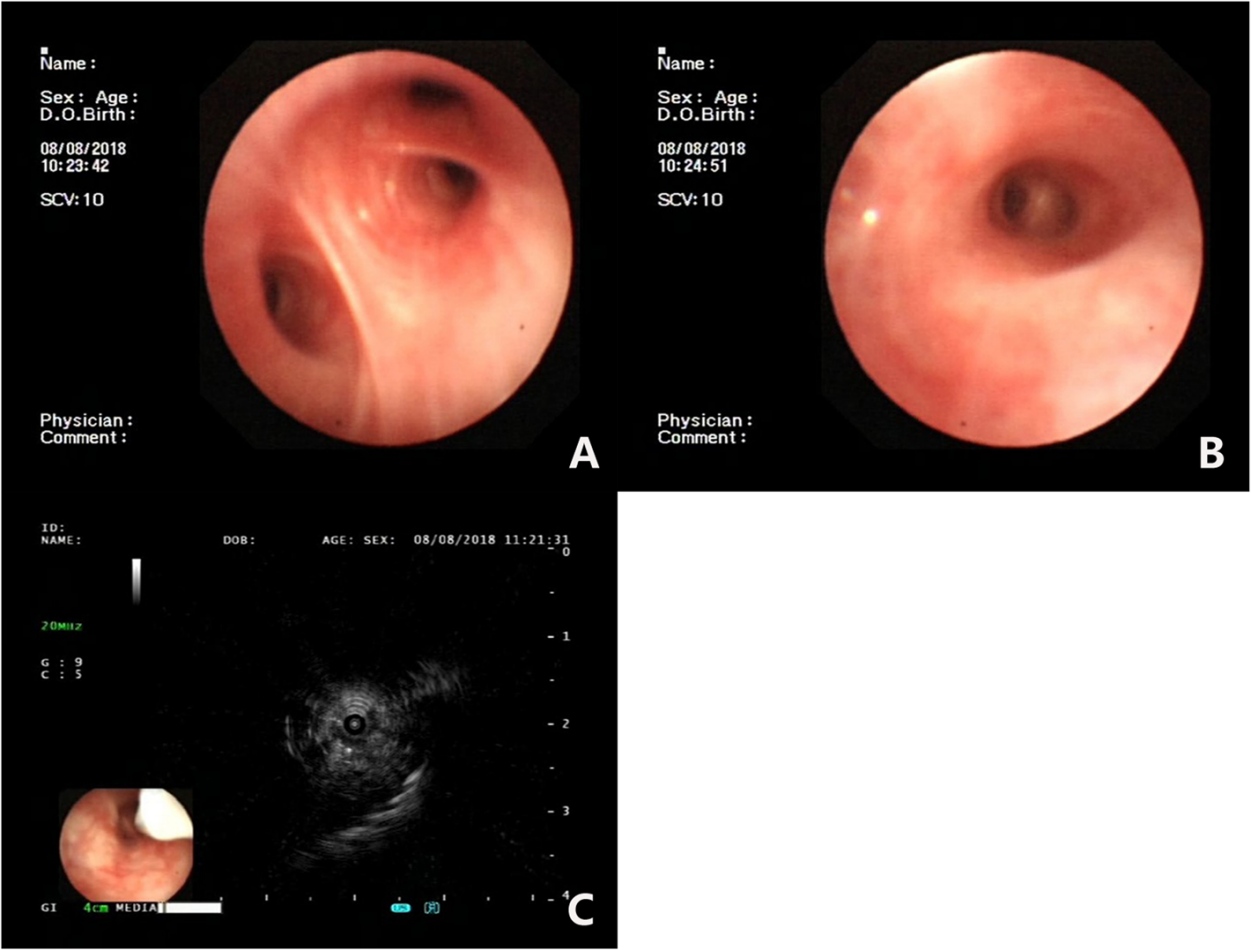
Bronchoscopy and EBUS of the lungs. **a** & **b**. The bronchi of right upper lobe showed clear patent lumen with mild congestion and edema, no neoplasia was detected. **c**. EBUS showed a focal hypoechogenic area with irregular boarder on the right B1a bronchi

**Fig. 3:**
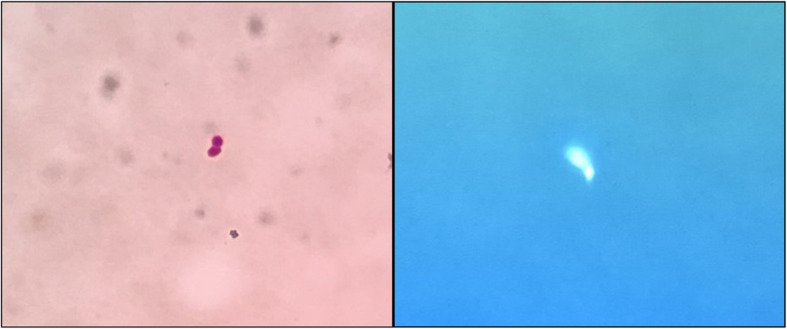
Gram stain and immunofluorescence of the lavage specimen showed the broad-based budding yeast of Blastomyces Dermatitidis

**Fig. 4:**
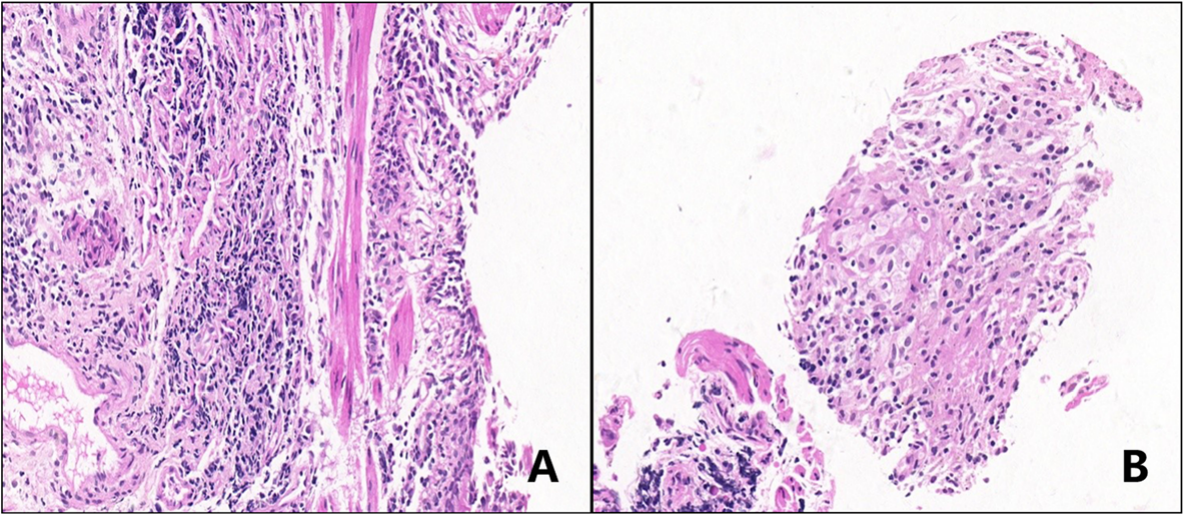
Histopathology analysis (40×) with H&E stain of the EBUS-TBLB sample. **a** Fibrous hyperplasia with abundant lymphocytes infiltration within the interstitial area, and visible alveolar extravasate. **b**.Fibrous exudates with moderate lymphocytes and plasma cells infiltration

Initial evaluation revealed that CBC, coagulation, electrolytes, liver, and kidney function were within normal limits. He did not undergo an image examination of his paranasal sinuses or immunoglobulin levels. He had bronchoscopy on the second day, and a hypoechogenic area at the right B1a bronchi was found (Fig. 2) on EBUS, followed by an EBUS-GS-TBLB. Samples of biopsy and BALF were sent for general analysis, Gram stain, histopathology, and NGS test. The general analysis of BALF showed predominant neutrophils and yeast-like fungi on smear. General fungal culture returned negative after 72 h incubation. However, NGS of both lavage and biopsy specimen revealed the presence of *Blastomyces dermatitidis*. Gram stain of the lavage specimen showed a gram-positive broad-based budding yeast, and immunofluorescence also revealed *Blastomyces dermatitidis* (Fig. 3). Histopathology was reported as chronic inflammation with fibrous exudates of the biopsied sample 1 week later (Fig. 4). The diagnosis was, therefore, confirmed.

**Fig. 5:**
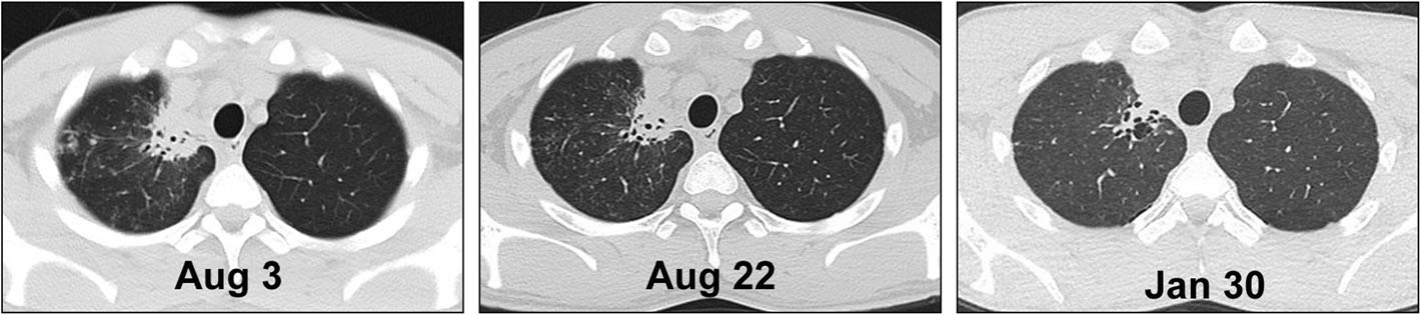
Follow-up CT scan of the lungs on August 22, 2018 and January 30, 2019. Absorbed exudation of the inflammatory lesion as the treatment continues, leaving small cavities and organic foci

He was treated with itraconazole 200 mg twice per day orally for 2 weeks, and chest CT scan showed continuously decreasing size of the lesion during the follow-up after 2 weeks (August 22, 2018) and after 5 months (January 30, 2019) (Fig. 5).

## Discussion and conclusion

Blastomycosis is a systemic pyogranulomatous infection that arises after inhalation of the conidia of the thermally dimorphic fungus *Blastomyces dermatitidis* or *Blastomyces gilchristii*. Following inhalation, conidia undergoes nonspecific phagocytosis and killing mediated by polymorphonuclear leukocytes (PMN), monocytes, and alveolar macrophages. Thus, the lungs are the most common site of infection [2]. Common symptoms include fever, non-productive cough, chest pain, and shortness of breath; more severe complications such as acute respiratory distress syndrome (ARDS) and respiratory failure tend to occur among immunocompromised population. Other uncommon extrapulmonary involvements such as weight loss, skin rash, and liver or kidney dysfunction are also described [3]. Chest radiography typically reveals alveolar infiltration, air-space like consolidation, or mass-like lesions, which may be misdiagnosed as acute bacterial pneumonia or lung malignancy. Chest CT scan may show pulmonary nodules, consolidation with or without cavitation, and/or tree-in-bud opacities. Small pleural effusions are frequent, and hilar adenopathy is rare [4].

The definitive diagnosis of blastomycosis requires the visualization of the organism on histology or a positive culture finding [5]. Typically, the yeast cells present single broad-based budding, recognized under Gram stain or periodic acid-Schiff (PAS) stain. More specific nucleic acid detection, including polymerase chain reaction (PCR) and repetitive DNA sequences such as NGS, tend to be more useful in complicated cases. Once the diagnosis is confirmed, anti-fungal treatment should be initiated as soon as possible. Options including amphotericin B or itraconazole and appropriate regimen must be decided upon the severity of disease and the immune status of the patient. Mild to moderate blastomycosis can be treated by oral itraconazole for 6–12 months and severe pulmonary or disseminated disease should be managed by intravenous lipid formulation of amphotericin B for at least 1 to 2 weeks [6] until improvement is observed, followed by oral itraconazole for at least 12 months.

Differential diagnosis should include opportunistic infections such as tuberculosis, histoplasmosis, and lung malignancy, considering our patient’s presentation. According to his history and limited symptoms, he grew up in China and moved to US a year ago, raising the suspicion of mycobacterium contact. However, initial lab evaluations showed negative results of sputum acid-fast stain and T-SPOT test. Although T-SPOT has a sensitivity of approximately 90 and > 95% on specificity [7], the negative predictive value is only 63.18% [8]. He underwent the first bronchoscopy, and the BALF analysis was negative for *Mycobacterium tuberculosis* DNA detection (Xpert), making TB the less likely diagnosis. The Xpert MTB/RIF test is a nucleic acid amplification test (NAAT) assay for the detection of *Mycobacterium tuberculosis* (MTB) and rifampin-resistance (RIF) mutations, which was approved by the FDA in 2013 because of the short testing time (within 2 h) and high sensitivity (89%) and specificity (99%) [9]. Other infections including histoplasmosis and non-TB mycobacterium should be carefully investigated as well. Histoplasmosis is caused by inhalation of the microconidia of *Histoplasma capsulatum*, which shares the same endemic area of *Blastomyces dermatitis*. Chest radiographs usually show enlarged hilar or mediastinal lymph nodes with focal patchy or nodular infiltrates; cavitation can also be seen resembling the reactivation of *Mycobacterium tuberculosis* infection. Therefore, obtaining the lesion sample and subsequent cytopathology and DNA sequence are essential to determine the etiology and further treatment.

On the second chest CT scan, the mass-like lesion was reported as lung malignant tumor. However, the tree-in-bud sign and thickening walls along the bronchioles and diffused ground glass appearance with doughy curved- spiculation suggested chronic inflammation other than a neoplasia in the lung. His young age, no family history of smoking or malignancy, and a lack of cachexia symptoms such as hemoptysis and weight loss made lung malignancy the inferior diagnosis. Consequently, we decided to perform the second bronchoscopy with pathology and cytology analysis of the lesion after obtaining his informed consent. The lesion was located at the apical segment of right upper lobe and no visible abnormalities were found under general bronchoscopy. As a result, we used EBUS and found a focal bulge with irregular boarder at the right B1a bronchi, which was detected as the hypoechogenic area, and biopsy sampling was obtained through EBUS-GS-TBLB. EBUS-GS-TBLB is most commonly used to sample pulmonary masses or nodules, endobronchial or peri bronchial lesions, as well as to guide therapeutic procedures. Through ultrasound detection, we ensured the accuracy of sampling to minimize the error of diagnosis, and the sample was sent for histopathology analysis and NGS test.

Since chronic infectious disease is the prior consideration of diagnosis and pulmonary tuberculosis has been excluded based on previous investigation, we preferred NGS as a method to identify the pathogen. NGS is a type of DNA sequencing technology that uses parallel sequencing of multiple small fragments of DNA to determine sequence. NGS plays a significant role not only in the diagnosis of genetic disease and malignancy but also in the diagnosis of unknown pathogens, outbreak infection investigations, identification of drug-resistant bacterial clones, and characterization of highly-virulent bacteria [10]. Although the cost of NGS is less economic and the results can be affected by the limited gene database, sample contamination, or incomplete workflow, it still has promising clinical usage because of its high sensitivity (less specimen requirements) and short turnaround time (3–5 days).

Our case presented a chronic atypical lung infection scenario and illustrated the importance of NGS usage in the diagnosis of a relatively rare or complex disease. Lack of typical manifestations of blastomycosis and low incidence in the non-endemic area of China might contribute to the delay in diagnosis. Though NGS is usually not essential to diagnose blastomycosis, in our case, it was very helpful since the traditional standard diagnostic assay have been failed to recognize the pathogen. After blastomycosis was confirmed according to NGS assay, the patient fully recovered after anti-fungal treatment. In conclusion, clinicians should consider chronic pulmonary blastomycosis when a patient presents with chronic lung infection with a travel history to an endemic area and should carefully differentiate it from other chronic infections and malignancy.

## Data Availability

All data generated or analyzed during this study are included in this published article. Besides, any additional data/files may be obtained from the corresponding author.

## Abbreviations

ARDS: Acute respiratory distress syndrome
BALF: Broncho alveolar lavage fluid
CT: Computed tomography
CBC: Complete blood count
WBC: White blood cells
EBUS-GS-TBLB: Endobronchial ultrasound-guide sheath-transbronchial lung biopsy
MTB: *Mycobacterium tuberculosis*
NAAT: Nucleic acid amplification test
NGS: Next generation sequencing
PMN: Polymorphonuclear leukocytes
PAS: Periodic acid-Schiff
PCR: Polymerase chain reaction
RBC: Red blood cells
RIF: Rifampin-resistance
